# The Correlation Between NLR, RDW, and Pulmonary Hypertension in Patients With Bronchiectasis and Chronic Obstructive Pulmonary Disease Overlap Syndrome

**DOI:** 10.1111/crj.70128

**Published:** 2025-09-29

**Authors:** Lingling Hu, Zhenxin Liu, Jiangtao Yu, Zhongfei Yang, Daxi Feng

**Affiliations:** ^1^ Department of Respiratory and Critical Care Medicine Qilu Hospital of Shandong University Dezhou Hospital Dezhou China; ^2^ Department of Critical Care Medicine Qilu Hospital of Shandong University Dezhou Hospital Dezhou China; ^3^ Department of Radiology Qilu Hospital of Shandong University Dezhou Hospital Dezhou China

**Keywords:** bronchiectasis and chronic obstructive pulmonary disease overlap syndrome, neutrophil to lymphocyte ratio, pulmonary hypertension, red blood cell volume distribution width

## Abstract

**Introduction:**

Based on the analysis of the relationship between neutrophil to lymphocyte ratio (NLR) and red blood cell distribution width (RDW) and pulmonary hypertension (PH) in patients with bronchiectasis and chronic obstructive pulmonary disease overlap syndrome (BCOS), this paper aims to explore the indexes that not only represent the severity of patients with BCOS overlapping PH but also are highly related to BCOS overlapping PH.

**Methods:**

The clinical data of 159 patients with BCOS admitted to Qilu Hospital of Shandong University Dezhou Hospital from January 2019 to November 2024 were collected and analyzed. All the patients had complete color Doppler echocardiography at this hospital and were separated into experimental group (106 cases, BCOS with PH) and control group (53 cases, BCOS not combined with PH group), according to whether they were complicated with pulmonary hypertension or not. And then the experimental group was divided into mild, moderate and severe subgroups. The correlation of NLR, RDW with pulmonary artery systolic blood pressure (PASP) in BCOS patients was analyzed. And whether there were differences or not between NLR and RDW among experimental group, control group as well as subgroups was compared. Furthermore, receiver operating characteristic (ROC) curves were constructed to evaluate the efficacy of NLR and RDW in distinguishing between “PH‐complicated” and “non‐PH‐complicated” statuses among BCOS patients at the cross‐sectional level.

**Results:**

First, the level of NLR and RDW in experimental group was higher than those in control group, in addition the difference was statistically significant (*p* < 0.05). Second, significant intergroup differences in NLR and RDW levels were observed among the three subgroups of the experimental group (NLR: *p* < 0.001; RDW: *p* = 0.011). Specifically, both NLR and RDW levels in the severe PH subgroup were significantly higher than those in the mild PH subgroup (NLR: adjusted *p* < 0.001; RDW: adjusted *p* = 0.009). Additionally, NLR levels in the severe PH subgroup were higher than those in the moderate PH subgroup (adjusted *p* = 0.011), whereas no statistically significant difference in RDW levels was noted between the severe and moderate PH subgroups (adjusted *p* = 0.148). Furthermore, there were no significant differences in NLR or RDW levels between the mild and moderate PH subgroups (NLR: adjusted *p* = 0.196; RDW: adjusted *p* = 0.607). Third, the level of NLR and RDW was positively correlated with PASP (*r* = 0.294, 0.259; *p* < 0.05). Fourth, Multivariate logistic regression analysis revealed that decreased PO_2_, NLR, and RDW are independent risk factors for PH development in BCOS patients (all *p* < 0.05). Fifth, ROC curve results showed the areas under the curve (AUC) of NLR, RDW and their combined detection in differentiating BCOS patients with and without PH were 0.628, 0.751, and 0.756 respectively. In particular, RDW performed better than NLR in differentiating with regard to discriminative ability. Furthermore, compared to RDW, the AUC of the combined detection was higher, meanwhile, its specificity was greatly enhanced than both single indicators. These results indicate that the combined detection exhibits better capability in identifying PH complicating BCOS at the cross‐sectional level.

**Conclusions:**

The level of NLR and RDW is related to the severity of pulmonary arterial pressure in patients with BCOS. The two indicators can serve as a significantly relevant factor for pulmonary hypertension complicating BCOS.

## Graphical abstract

The levels of neutrophil to lymphocyte ratio (NLR) and red blood cell volume distribution width (RDW) are related to the severity of pulmonary arterial pressure in patients with bronchiectasis‐chronic obstructive pulmonary disease overlap syndrome (BCOS). The two indicators can serve as significantly relevant factors for pulmonary hypertension complicating BCOS.

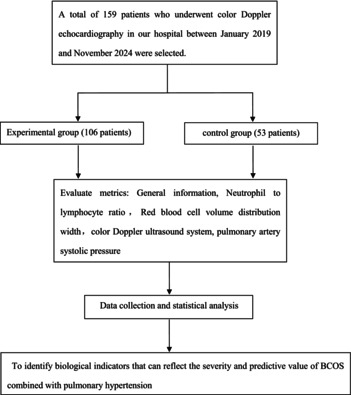

## 1 Introduction

Bronchiectasis is a chronic respiratory condition characterized by recurrent suppurative infections. The etiology of this disease is multifactorial, encompassing infectious agents, immune system dysfunctions, and genetic predispositions. Repeated damage and/or obstruction of the small and medium bronchi leads to structural destruction of the bronchial walls, ultimately resulting in chronic bronchiectasis. Patients frequently experience chronic cough, excessive sputum production, and may develop intermittent hemoptysis; in severe cases, this can progress to respiratory failure. Chronic obstructive pulmonary disease (COPD) is a heterogeneous lung disorder characterized by abnormalities in the airways (such as bronchitis and bronchiolitis) and/or alveoli (such as emphysema), often marked by persistent and progressive airflow limitation.

It is previously believed that bronchiectasis and chronic obstructive pulmonary disease (COPD) are distinct and independent conditions. However, recent studies have revealed a significant overlap between bronchiectasis and COPD, with the prevalence of bronchiectasis in COPD patients ranging from 26% to 69% [1]. In various studies, the prevalence of bronchiectasis and chronic obstructive pulmonary disease overlap syndrome (BCOS) has been reported to range from 4% to 72%, which may be attributed to differences in study populations and inclusion criteria [2, 3]. Patients with BCOS exhibit a poorer prognosis and higher mortality compared to those with either condition alone [4, 5]. For BCOS, the BRONCH‐UK consortium has established its diagnostic criteria; however, the pathogenesis of BCOS remains unclear. Studies have demonstrated that potential pathogenic microorganisms, particularly *Pseudomonas aeruginosa* isolated from the airways, are associated with BCOS [6]. Long‐term smoking is a significant risk factor for BCOS, likely due to chronic airway inflammation induced by tobacco exposure.

Pulmonary hypertension (PH) is a condition characterized by alterations in pulmonary vascular structure and function, resulting from various etiologies. Without timely intervention, PH can progressively worsen, ultimately leading to chronic cor pulmonale, which manifests as right ventricular dysfunction. This condition often has a poor prognosis and is associated with significantly increased mortality. Studies have demonstrated that the incidence of pulmonary hypertension is high in patients with COPD [7], and bronchiectasis also represents a risk factor for vascular complications. In patients with BCOS, concurrent damage to the lung parenchyma and airway walls results in impaired pulmonary ventilation and gas exchange, leading to destruction of the vascular bed and hemodynamic changes [8].

Currently, limited research has been conducted on BCOS combined with PH, and the underlying mechanisms remain unclear. Patients with BCOS who are accompanied by pulmonary arterial hypertension usually have a poorer prognosis. Therefore, early diagnosis and treatment of BCOS combined with PH are crucial for improving patient outcomes. In light of this, this study investigates the correlation between peripheral serum neutrophil‐to‐lymphocyte ratio (NLR) and red cell distribution width (RDW) levels in patients with BCOS combined with PH. The findings offer valuable clinical insights for the early detection and assessment of PH in BCOS patients.

## 2 Methods

### 2.1 Subjects and Grouping

Clinical data were collected and analyzed from 159 patients diagnosed with BCOS admitted to the Department of Respiratory and Critical Care Medicine, Qilu Hospital of Shandong University Dezhou Hospital, between January 2019 and November 2024. All patients underwent color Doppler echocardiography in our hospital using a Philips EPIQ CVx color Doppler ultrasound system, performed by sonographers with at least 5 years of work experience. Pulmonary artery systolic pressure (PASP) was measured via the tricuspid regurgitation velocity method, with the calculation formula: PASP = 4 × V^2^ + estimated right atrial pressure (according to the 2015 European Respiratory Society/European Society of Cardiology [ERS/ESC] guidelines, right atrial pressure was set to a default value of 5–10 mmHg). Each patient was measured consecutively 3 times, and the mean value was taken as the final result. Patients were categorized into two main groups based on the presence or absence of PH: the BCOS combined with PH group (106 cases) and the control group (53 cases, BCOS without PH). The BCOS combined with PH group was further subdivided into three categories based on pulmonary artery systolic pressure (PASP): mild (31 cases, 36 mmHg ≤ PASP < 50 mmHg), moderate (45 cases, 50 mmHg ≤ PASP < 70 mmHg), and severe (30 cases, PASP ≥ 70 mmHg).

### 2.2 Rationale for Sample Size Determination

Based on the HIS, a total of 205 patients with probable cases diagnosed with BCOS between January 2019 and November 2024 were found initially. Through stringent screening according to inclusion and exclusion criterions, 46 cases were excluded because of lack of baseline data, comorbid conditions with other underlying diseases that may lead to PH, and other reasons. In total, 159 eligible cases were eventually enrolled. Sample size statistical power was verified using post hoc analysis with G*Power 3.1 software. For the primary outcome indicator, that is, the correlation between NLR and the severity of pulmonary hypertension in BCOS patients, referring to the research results of Zuo et al. [9], the effect size (Cohen's *f* = 0.268) was adopted. With a two‐tailed α set at 0.05, the statistical power of the sample size of 159 cases was calculated to be 85.6%, which was higher than the accepted standard of 80%. For the other primary outcome indicator, that is, the correlation between RDW and the severity of pulmonary hypertension in BCOS patients, referring to the findings of Yang et al. [10], the effect size (Cohen's *f* = 0.585) was used. With a two‐tailed α set at 0.05, the statistical power of 159 cases was calculated to be 99%, which was significantly higher than the 80% threshold. These results indicated that the sample size in this study was sufficient to detect the expected intergroup differences.

The sample size of this study (159 cases) was comparable to that of published retrospective studies in the same field (with sample sizes ranging from 78 to 213 cases) [9, 10]. The sample size of each subgroup was ≥ 30 cases, which met the basic statistical requirements for Kruskal–Wallis test and Bonferroni‐corrected pairwise comparisons.

### 2.3 Inclusion and Exclusion Criteria

Inclusion Criteria: (1) All patients had complete inpatient medical records; (2) Patients met the diagnostic criteria for BCOS established by the BRONCH‐UK consortium, with independent diagnosis by two experienced clinicians. In cases of disagreement, consensus was reached through discussion. The diagnostic criteria are as follows: ① Chronic obstructive pulmonary disease (COPD): presence of physiological features of irreversible airflow limitation, that is, forced expiratory volume in 1 s/forced vital capacity (FEV_1_/FVC) < 0.7 and FEV_1_% predicted < 80% after inhalation of bronchodilators, caused by abnormal inflammatory responses of the lungs to harmful stimuli (most commonly cigarette smoke). ② Bronchiectasis: Structural changes of airway dilatation and airway wall thickening confirmed by imaging. Chest computed tomography (CT) shows a bronchus‐to‐artery diameter ratio > 1 and airway wall thickening ≥ 1 mm. A diagnosis of BCOS requires simultaneous fulfillment of the above physiological criteria for COPD and structural criteria for bronchiectasis. (3) PH was diagnosed using the ultrasonic evaluation criteria outlined in the 2015 Guidelines for the Diagnosis and Treatment of Pulmonary Hypertension jointly issued by the European Respiratory Society (ERS) and the European Society of Cardiology (ESC). Exclusion Criteria: (1) Other diseases that may cause pulmonary hypertension, such as chronic thromboembolic pulmonary hypertension, interstitial lung disease, and portal hypertension; (2) Severe pneumonia with concurrent systemic infections; (3) Patients with PH who were treated with targeted therapies; (4) Patients with acute cardiovascular or cerebrovascular diseases, or hepatic and renal insufficiency;(5)Patients who had received anti‐inflammatory medications (e.g., glucocorticoids, immunosuppressants, or non‐steroidal anti‐inflammatory drugs) within 3 months prior to admission. This study has been approved by the Ethics Committee of Dezhou Hospital, Qilu Hospital of Shandong University.

### 2.4 Data Collection

Clinical and laboratory parameters were collected for all patients at admission, including age, gender, BMI, smoking history, comorbidities (e.g., hypertension, diabetes mellitus, coronary heart disease), as well as blood routine parameters, absolute neutrophil count (NEUT), absolute lymphocyte count (LYMPH), red blood cell distribution width (RDW), pulmonary artery systolic pressure (PASP), D‐dimer, and partial pressure of oxygen (PO_2_). The neutrophil‐to‐lymphocyte ratio (NLR) was calculated as NEUT/LYMPH. Pulmonary hypertension (PH) was defined by PASP measured via Philips EPIQ CVx color Doppler ultrasound.

### 2.5 Statistical Analysis

Statistical analysis was performed using SPSS 27.0 software. Measurement data that followed a normal distribution were expressed as mean ± standard deviation (X ± S). Comparisons between two groups were conducted using independent samples *t*‐tests, while multi‐group comparisons were analyzed using one‐way analysis of variance (ANOVA). For non‐normally distributed data, a non‐parametric rank sum test was employed for analysis, and the results were presented as median (P25, P75). Correlation analysis was conducted using Spearman's rank correlation test. Logistic regression analysis was employed to identify factors influencing pulmonary hypertension (PH) in patients with bronchiectasis‐chronic obstructive pulmonary disease overlap syndrome (BCOS). Receiver operating characteristic (ROC) curves were constructed to evaluate the predictive value of each index for PH in BCOS patients. The difference was statistically significant (*p* < 0.05).

## 3 Results

### 3.1 Comparison of Clinical Data Between the Experimental Group and the Control Group

There were no significant differences in age, gender, body mass index (BMI), smoking history, and comorbidities (hypertension, diabetes mellitus, coronary heart disease) between the two groups (*p* > 0.05). Compared with the control group, the experimental group exhibited lower partial pressure of oxygen (PaO_2_) (*p* < 0.05), while D‐dimer levels, neutrophil‐to‐lymphocyte ratio (NLR), and erythrocyte distribution width (RDW) were significantly higher (*p* < 0.05). Detailed results are presented in Table 1.

**TABLE 1:** Comparison of clinical data between the experimental group and control group.

Items	Trial group (*n* = 106)	Control group (*n* = 53)	x^2^/Z/t	*p*
Age (years)	67.83 ± 9.75	68.92 ± 9.53	−0.672	0.503
Gender (male/female)	(46/60)	(29/24)	1.817	0.178
BMI (kg/m^2^)	22.44 ± 3.13	23.00 ± 2.10	−1.338	0.183
Smoking history (with/without)	(29/77)	(22/31)	3.248	0.072
Hypertension (with/without)	(75/31)	(32/21)	1.729	0.189
Diabetes mellitus (with/without)	(97/9)	(45/8)	1.614	0.204
Coronary heart disease (with/without)	(61/45)	(36/17)	1.600	0.206
PO_2_ (mmHg)	69.50 (59.00, 81.00)	83.00 (76.00, 90.00)	−4.914	< 0.001
D‐dimer (μg/L)	246.00 (140.25, 458.75)	157.00 (89.50, 303.50)	−3.102	0.02
NLR	5.27 (3.45, 8.38)	3.58 (2.42, 7.01)	−2.622	0.009
RDW (%)	13.80 (13.10, 15.10)	12.80 (12.50, 13.40)	−5.146	< 0.001

Abbreviations: BMI, body mass index; NLR, neutrophil to lymphocyte ratio; PO_2_, the partial pressure of oxygen; RDW, red blood cell volume distribution width.

### 3.2 Comparison of NLR and RDW Among the Three Subgroups in the Experimental Group

The Kruskal–Wallis test was performed to examine the levels of NLR and RDW in three subgroups of the experimental group (mild, moderate, and severe PH groups). The result indicated that between groups had significance (NLR: *p* < 0.001; RDW: *p* = 0.011). In order to identify the significance of difference in intergroup, pairwise comparisons were made using Bonferroni correction. The findings showed that: the levels of the severe PH group were statistically higher than those in the mild PH group (NLR: adjusted *p* < 0.001; RDW: adjusted *p* = 0.009), the NLR level in the severe group was higher than that in the moderate group (NLR: adjusted *p* = 0.011); however, the RDW levels in the severe group and moderate group have no significant difference (RDW: adjusted *p* = 0.148). Moreover, we did not find any major differences in the levels of NLR or RDW between the mild and moderate PH groups (NLR: adjusted *p* = 0.196; RDW: adjusted *p* = 0.607). Detailed results are presented in Table 2.

**TABLE 2:** Comparison of NLR and RDW among three subgroups stratified by pulmonary hypertension severity.

	NLR	RDW (%)
Light PH group (*n* = 31)	3.41 (2.61, 6.93)	13.30 (12.90, 14.00)
Moderate PH group (*n* = 45)	4.68 (3.57, 7.25)	13.80 (12.80, 15.50)
Heavy PH group (*n* = 30)	7.33 (5.15, 17.10)	14.05 (13.50, 16.35)
H	19.290	8.981
*p*	< 0.001	0.011

*Note:* Comparisons among three groups were performed using Kruskal–Wallis test. Bonferroni correction was applied for multiple pairwise comparisons, with statistical significance set at *p* < 0.017.

Abbreviations: NLR, neutrophil‐to‐lymphocyte ratio; PH, pulmonary hypertension; RDW, red blood cell volume distribution width.

### 3.3 Analyze the Correlation Between NLR, RDW, and PASP

Correlation Analysis of NLR, RDW, and PASP: The correlation analysis revealed that NLR was positively correlated with PASP (*r* = 0.294, *p* = 0.002), and RDW was also positively correlated with PASP (*r* = 0.259, *p* = 0.007).

### 3.4 Factors Associated With PH in Patients With BCOS

Statistically significant clinical indicators (PaO_2_, D‐dimer, NLR, RDW) were set as independent variables, while the presence of PH in BCOS patients was set as the dependent variable. Multivariate logistic regression analysis was conducted. The results indicated that PaO_2_, NLR, and RDW were independent risk factors for PH in BCOS patients (*p* < 0.05). Detailed results are presented in Table 3.

**TABLE 3:** Logistic regression analysis of factors influencing concurrent PH in BCOS patients.

Variables	Univariate analysis			Multivariate analysis		
	OR	95% CI	*p*	OR	95% CI	*p*
PO_2_ (mmHg)	0.973	0.957–0.990	0.002	0.978	0.959–0.996	0.018
D‐dimer (mg/L)	1.002	1.000–1.003	0.039	1.001	0.999–1.002	0.422
NLR	1.171	1.051–1.304	0.004	1.134	1.015–1.268	0.027
RDW (%)	1.830	1.310–2.556	< 0.001	1.611	1.141–2.274	0.007

Abbreviations: CI, confidence interval; NLR, neutrophil to lymphocyte ratio; OR, odds ratio; PO_2_, the partial pressure of oxygen; RDW, red blood cell volume distribution width.

### 3.5 Discriminative Efficacy of NLR, RDW, and Their Combination for BCOS Complicated With PH

**FIGURE 1:**
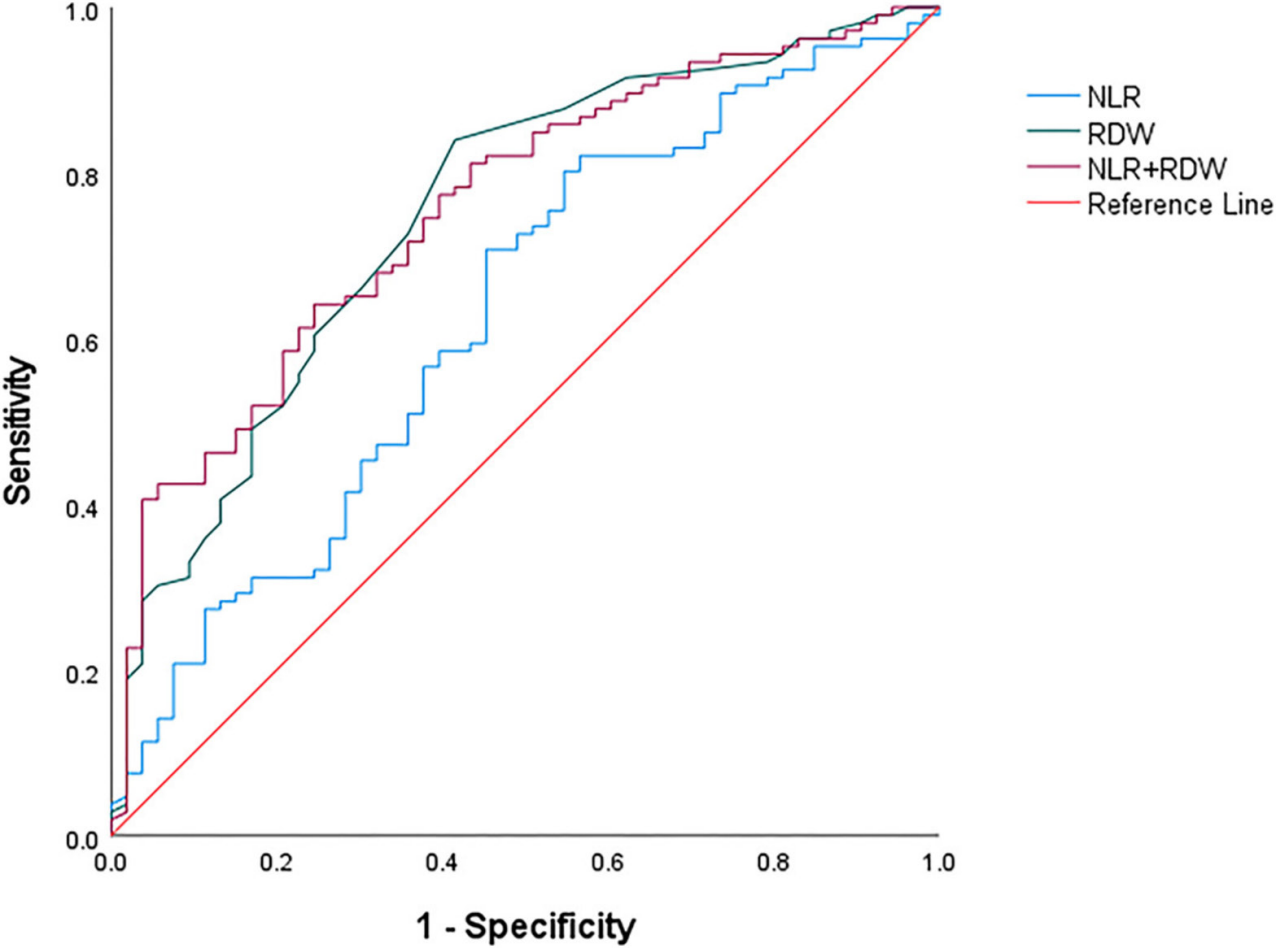
Evaluation of the predictive value of NLR, RDW, and their combination for BCOS complicated with PH using ROC curve analysis.

ROC curve analysis demonstrated that the areas under the curve (AUC) of NLR, RDW, and their combined detection for differentiating BCOS patients with and without PH were 0.628, 0.751, and 0.756, respectively. Specifically, RDW alone had better discriminatory power than NLR, and the AUC of combined detection was slightly higher than that of RDW alone, while it had better specificity compared with the two single indexes. These results indicate that combined detection has greater ability to detect PH in BCOS patients in a cross‐sectional manner. Detailed results are presented in Table 4 and Figure 1.

**TABLE 4:** The diagnostic discriminative efficacy of NLR, RDW levels, and their combination for distinguishing BCOS with PH from BCOS without PH.

Groups	Cutoff	AUC	95% CI	Sensitivity (%)	Specificity (%)	*p*
NLR	3.73	0.628	0.535–0.721	0.708	0.547	0.009
RDW (%)	12.85	0.751	0.670–0.831	0.840	0.585	0.000
Combined testing	—	0.756	0.679–0.834	0.642	0.755	0.000

Abbreviations: AUC, area under the curve; CI, confidence interval; NLR, neutrophil to lymphocyte ratio; RDW, red blood cell volume distribution width.

## 4 Discussion

The neutrophil‐to‐lymphocyte ratio (NLR) is a recognized biomarker of systemic inflammation and stress response. As a comprehensive indicator, NLR provides a more accurate evaluation of the systemic inflammatory response and disease prognosis compared to white blood cell (WBC) count, lymphocyte count, and neutrophil count. Owing to its advantages of easy accessibility, non‐invasive procedures, and cost‐effectiveness, this method has been extensively utilized in clinical research. Previous studies have demonstrated the prognostic value of NLR in patients with various solid tumors [11], particularly in those with lung cancer [12, 13, 14]. Moreover, NLR has also been shown to possess significant prognostic utility in several non‐neoplastic conditions, including pulmonary embolism [15], severe trauma [16], SARS‐CoV‐2 infection [17], and septic shock [18]. By analyzing the NLR values and survival times of hospitalized patients with acute exacerbation of chronic obstructive pulmonary disease (AECOPD) and discharged patients in the stable phase of COPD, it was demonstrated that the NLR value increases with the severity of the condition in patients with COPD [19]. By comparing the NLR levels of patients with chronic obstructive pulmonary disease and bronchiectasis, it was found that the NLR level was higher in the chronic obstructive pulmonary disease group. The study concluded that NLR is significant for assessing both COPD and bronchiectasis [20]. Additionally, some studies have shown that NLR levels are elevated in patients with pulmonary hypertension compared to healthy control, indicating that NLR has value in evaluating AECOPD with pulmonary hypertension and assessing the severity of pulmonary hypertension [9].

It has been reported that RDW is associated with inflammatory response, oxidative stress, and tumor necrosis factor‐α (TNF‐α) [21] and may serve as a robust predictor of various diseases, including acute ischemic stroke [22], acute pancreatitis [23], and sepsis [24]. Studies have indicated that RDW is an independent risk factor for COPD complicated with pulmonary hypertension [10], and this indicator is positively correlated with the severity of pulmonary hypertension in patients with COPD. RDW may also be a powerful predictor of prognosis in patients with COPD complicated by pulmonary hypertension [25]. Research has shown that the incidence of bronchiectasis combined with PH and cardiac insufficiency is approximately 31% [26]. The primary pathophysiological mechanism may involve hypoxia‐induced pulmonary vasoconstriction, leading to increased pulmonary vascular resistance, elevated pulmonary artery pressure, and ultimately right heart failure. To date, no studies have specifically examined the relationship between RDW and bronchiectasis combined with PH.

This present study demonstrated that NLR and RDW were significantly higher in BCOS patients with PH, and both were positively correlated with the PASP. The levels of these markers were most elevated in the severe PH group, and the results of further multivariate regression analysis demonstrated that the NLR and RDW were independent risk factors for the patients with PH complicated by BCOS. One of the essential reasons why this is possible is the “complicated pathological features” of BCOS: ① Cause of high NLR: The main inflammatory cells of bronchiectasis and COPD are neutrophils and T lymphocytes. Neutrophils release the proteases and cause bronchial structural destruction, and T lymphocytes promote the enlargement of peripheral bronchial lymph node and chronic bronchitis [6, 27]. In the inflammatory process, the neutrophil‐to‐lymphocyte ratio (NLR) rises owing to lymphocyte apoptosis and an increase in neutrophil counts (as phagocytes). The hypothesis for the increase in NLR in BCOS patients was that there was more significant airflow restriction and higher pulmonary artery pressure than that in COPD patients without bronchiectasis, due to the inflammatory response. This causes persistent airway injury, which contributes to a worse lung function and also rapid disease progression in BCOS patients [28]. The levels of serum NLR in BCOS patients with PH were higher than those without PH, and were positively associated with the severity of PH in BCOS patients, and suggested a more powerful systemic inflammatory response in vivo. ② The mechanism of elevated RDW: In pulmonary diseases, the mechanism of PH of chronic pulmonary origin lies in two main aspects: First, pulmonary infection chronically changes the structure of the pulmonary interstitium, which impacts lung gas exchange under normal conditions and causes tissue hypoxia. Secondly, this hypoxic state also triggers pulmonary vasoconstriction; Additionally, the pulmonary destruction itself causes hemodynamic changes, subsequently leading to insufficient blood perfusion. These pathological changes cause resistance in the pulmonary circulation, increased pulmonary artery pressure, right heart overload and subsequently right heart failure [29]. In contrast, compared with BCOS patients without PH, those with PH have a stronger inflammatory response and obvious tissue hypoxia, releasing large amounts of inflammatory factors, producing increased oxygen free radicals, and triggering the activation of the complement system. The activation of the complement system and aggravated inflammatory response will damage red blood cell deformability, resulting in abnormal changes in the red blood cell volume. Hence, this leads to a major increase in RDW values in BCOS patients with PH.

In the current study, RDW values of the severe PH group were higher than those of the mild PH group, but there was no significant difference from the moderate PH group. The possible reason may be some degrees of erythropoiesis disorder in the moderate PH group or the relatively small number of the moderate group (*n* = 45). It indicates that RDW has poor discriminative power in differentiating moderate and severe PH; there might be a threshold phenomenon in its relationship with pulmonary vascular remodeling, which needs to be confirmed by large‐sample studies.

The intermediate AUC obtained for NLR (AUC = 0.628), RDW (AUC = 0.751) and their combination (AUC = 0.756) in our study can be justified by the fact that both are non‐specific inflammatory/hypoxia markers and do not capture all pathophysiological aspects of BCOS‐PH (e.g., pulmonary vascular remodeling, endothelial dysfunction). We still note an AUC for RDW similar to that of biomarkers for COPD‐associated PH presented in other studies [10]. Moreover, the relatively high specificity of the combination (75.5%) confirms that these two routine parameters should be applied as a screening tool to rapidly identify high‐risk patients within primary health care, thus avoiding missed diagnoses of PH. The relatively low synergistic combination effect might be caused by partially shared information between NLR and RDW as these two parameters share an “inflammation‐hypoxia” pathway. In the future, the pulmonary function parameters (e.g., DLCO, FEV1 and/or PEF) could be added to the combination; indeed, those parameters, like the blood parameters, have shown great value in PH screening [7, 8]; the change of FEV_1_% predicted and the CT change of vascular remodeling could be applied to establish the multi‐dimensional model that could enhance the clinical value for identification.

In conclusion, NLR and RDW share partial correlations with complex BCOS complicated with PH. As routine indexes routinely tested in blood, NLR and RDW possess merits like simplicity and low cost and show superiority when combined as an effective test to screen for PH complicated with complex BCOS. In primary hospitals, their central significance should lie in providing primary help in early PH detection and stratification from routine blood tests—a role that leads to further echocardiography, or adjustments to interventions (e.g., optimizing anti‐inflammatory treatment, improving oxygenation), which can greatly contribute to further improvements in patients' prognosis and quality of life.

The drawbacks of this study are: (1) The single‐center retrospective design makes it prone to selection bias. Additionally, the completeness and accuracy of data on clinical variables (including disease duration and frequency of acute exacerbations) are limited, and these variables were not included in the multi‐factor model for strict control, which may have indirectly affected the results; (2) In this study, the identification of pulmonary hypertension (PH) was based exclusively on echocardiographic estimation of pulmonary artery systolic pressure (PASP), rather than the definitive diagnostic method of right heart catheterization (RHC). It is recognized that this approach has certain drawbacks.

However, the independent correlations of NLR and RDW observed in the multi‐factor regression analysis, as well as the consistent trend between their levels and PH grades, indicate that the main findings of the present study are somewhat robust. Future research may further elucidate the temporal sequence between changes in their levels and the onset of PH through prospective cohort studies, confirm the results using right heart catheterization, and strictly control confounders such as disease duration and frequency of acute exacerbations to further enhance their clinical application value.

## Author Contributions

Lingling Hu: Experimental design, article writing; Zhenxin Liu: Statistical analysis and mapping of data; Jiangtao Yu: Data collation; Zhongfei Yang, Daxi Feng: research guidance, paper review. All authors read and approved the final manuscript.

## Ethics Statement

This study protocol was reviewed and approved by Qilu Hospital of Shandong University Dezhou Hospital, approval number 2024154. The study has been granted an exemption from the requirement for written informed consent by Dezhou Hospital of Qilu Hospital of Shandong University.

## Conflicts of Interest

The authors declare no conflicts of interest.

## Data Availability

The data that support the findings of this study are not publicly available due to their containing information that could compromise the privacy of research participants but are available from the corresponding author Z.Y. upon reasonable request.
